# Bowel and bladder function in infant toilet training (BABITT) – protocol for a randomized, two-armed intervention study

**DOI:** 10.1186/s12887-022-03355-6

**Published:** 2022-05-19

**Authors:** Terese Nilsson, Anna Leijon, Ulla Sillén, Anna-Lena Hellström, Barbro Hedin Skogman

**Affiliations:** 1 Department of Family medicine and Center for Clinical Research Dalarna - Uppsala University, Region Dalarna County, Falun, Sweden; 2 Department of Medicine and Health Sciences, Örebro University, Örebro, Sweden; 3 Department of Pediatric Surgery, Pediatric Uronephrologic Centre, Queen Silvia Children’s Hospital, Institute of Clinical Sciences, Sahlgrenska Academy, University of Gothenburg, Göteborg, Sweden; 4 Institute of Health and Care Sciences, University of Gothenburg, Göteborg, Sweden; 5 Department of Pediatrics and Center for Clinical Research Dalarna - Uppsala University, Region Dalarna County, Falun, Sweden

**Keywords:** Assisted infant toilet training, Children, Infant colic, Infant dyschezia, Functional constipation, Bladder dysfunction, Stool toileting refusal, Child health care, Infant-to-mother attachment, Parental stress

## Abstract

**Background:**

In the last decades, the average age for toilet training has increased in the western world. It is suggested that the postponed initiation of toilet training is a contributing factor to problems related to bowel and bladder control. Functional gastrointestinal and urinary tract disorders are prevalent in childhood, causing suffering in affected children and for their families, and consuming healthcare resources. To evaluate whether assisted infant toilet training can prevent functional gastrointestinal and urinary tract disorders in young children, we are conducting a randomized intervention study with a 4-year follow-up.

**Methods:**

This randomized two-armed intervention study will include 268 Swedish infants recruited at six child healthcare centers in Region Dalarna located in the central part of Sweden. The intervention entails parents being instructed and practicing assisted infant toilet training with their child. Children are randomized to start assisted infant toilet training at 0–2 months or at 9–11 months of age.

The primary objective is to determine the efficacy of assisted infant toilet training initiated at 0–2 months on the prevalence of functional gastrointestinal disorders (defined as infant colic, infant dyschezia and/or functional constipation) up to the age of 9 months. Secondary objectives are to evaluate whether assisted toilet training initiated during the first year of life reduce the prevalence of functional gastrointestinal disorders (defined as functional constipation, gastrointestinal symptoms and/or stool toileting refusal) and urinary tract disorders (defined as bladder dysfunction and/or urinary tract infections) up to the age of 4 years. Furthermore, infant-to-mother attachment, parental stress, the toilet training process and overall parental experiences will be evaluated/explored.

**Discussion:**

This protocol article presents the rationale and design of a randomized two-armed intervention study that will determine the efficacy of assisted infant toilet training on functional gastrointestinal disorders up to the age of 9 months. Furthermore, the study will evaluate whether assisted infant toilet training during the first year of life can prevent functional gastrointestinal and urinary tract disorders in children up to 4 years of age. If effective, assisted infant toilet training could be recommended in child healthcare settings and new evidence-based guidelines on infant toilet training could be implemented.

**Trial registration:**

The study protocol was retrospectively registered at ClinicalTrials. gov  (NCT04082689), initial release June 12th, 2019)

## Background

In the last decades, the average age for toilet training has increased in the western world [1]. In the 1960’s the vast majority of Swedish children were regularly using the potty by their first birthday [2]. In contrast, being in diapers at the age of three is not unusual today [3]. It is suggested that the postponed initiation of toilet training is a contributing factor to problems related to bowel and bladder control [4–9].

In Vietnam, toilet training starts in early infancy by tradition. By the age of 9 months, the majority of children achieve parent-assisted dryness [10]. In the western world, this technique for early toilet training has received attention, referred to by the terms “Elimination Communication” or “Assisted Infant Toilet Training”. As the parent responds to the elimination signals and routines of the child by taking it to a designated place to relieve itself rather than in a diaper, the baby’s cues and ability to convey elimination needs are gradually enhanced. Through this responsive interaction, a two-way communication pathway develops between the parent and the child [11]. In infant toilet training, the baby is held in a squat position over some sort of receptacle (e.g. potty or basin). The squat position relaxes the pelvic floor [12] and thus improve the emptying ability of the urinary bladder, but probably also facilitate fecal evacuation, the latter shown in adults [13].

The Swedish National Guide to Child Healthcare is web-based decision support, providing evidence-based guidelines for Swedish child healthcare centers. In 2015, a new recommendation was published, stating that initiation of toilet training should be encouraged, at the latest, at the 10-month visit at the child healthcare center and preferably before the child can walk [14].

Infant colic is characterized by inconsolable crying or fussing without obvious cause in infants under the age of 5 months. A systematic review found that the overall prevalence of infant colic ranged from 17 to 25% during the first 6 weeks of life [15]. Infant colic is a functional gastrointestinal disorder that often causes great stress for the family while it is ongoing, despite its benign and self-limited character. The cause of infant colic is unknown [16].

Infant dyschezia is a functional condition in children less than 9 months old defined by at least 10 minutes of straining and crying before successful or unsuccessful passage of soft stools [17]. Prevalence varies from 0.9–3.9% in children < 9 months [17]. The cause is considered to be dyscoordination between intra-abdominal pressure and relaxation of the pelvic floor muscles [17].

In the newborn, incomplete voiding and residual urine is normal. This is caused by dyscoordination between the expelling bladder muscle and the closing sphincter. As shown by a study on Vietnamese and Swedish children [18], coordination develops in association with toilet training and not through passive maturation, as previously prevailing opinion has suggested. The study showed a prominent difference, the Vietnamese infants (with parents practicing assisted infant toilet training) had no residual urine at the age of 9 months, compared with the Swedish children (without parents practicing assisted infant toilet training), who showed complete emptying of the bladder at the age of 36 months [18]. Symptoms of bladder dysfunction are common in school-age children and most often present as urgency and incontinence [19–21]. Furthermore, functional constipation is known to negatively affect bladder function, which is supported by the fact that symptoms of bladder dysfunction as well as recurrent urinary tract infections often disappear with the treatment of concomitant constipation [22].

The prevalence of constipation, as presented in international studies, is very variable (0.7–30%), possibly due to the multifactorial nature of the condition, but also due to the lack of uniform definitions [23]. According to preliminary results from an ongoing Swedish study, the prevalence of functional constipation is estimated to 7% during the first year of life (personal communication). In most cases of children with constipation (> 90%), the condition is defined as functional constipation, i.e. no organic cause is found [23]. Despite good treatment options, it has been shown that quality of life in constipated children is significantly decreased compared to children without constipation [24, 25]. Furthermore, children often suffer relapses during childhood and up to a third still experience symptoms of constipation in adulthood [26–28].

In summary, functional gastrointestinal and urinary tract disorders are common, cause suffering for affected children and their families, and consume healthcare resources. To evaluate whether assisted infant toilet training can prevent functional gastrointestinal and urinary tract disorders in young children, we conduct a randomized intervention study with a 4-year follow-up and the study protocol is outlined below.

### Objectives and research hypothesis

The primary objective of this study is to determine the efficacy of assisted infant toilet training initiated at 0–2 months on the prevalence of functional gastrointestinal disorders (defined as infant colic, infant dyschezia and/or functional constipation) up to the age of 9 months. We hypothesize that assisted infant toilet training significantly reduces the prevalence of functional gastrointestinal disorders up to the age of 9 months.

Secondary objectives are to evaluate whether assisted infant toilet training initiated during the first year of life reduces the prevalence of functional gastrointestinal disorders (defined as functional constipation, gastrointestinal symptoms and/or stool toileting refusal), and urinary tract disorders (defined as bladder dysfunction and/or urinary tract infections) up to 4 years of age. Furthermore, infant-to-mother attachment, parental stress, the toilet training process, and the overall parental experiences will be evaluated/explored.

## Methods/design

### Study design and setting

The BABITT-study is designed as a randomized, investigator-blinded, two-armed intervention study with a 4-year follow-up. The study protocol is adhering to the SPIRIT 2013 statement [29] and the full clinical study protocol is available at ClinicalTrials.govNCT04082689. Report of results from the study will follow the CONSORT statement [30].

The study is conducted at six child healthcare centers in an urban-rural setting in Region Dalarna, in central Sweden. The child healthcare centers are chosen for geographical (urban and rural), socioeconomic, but also operational and logistical purposes. The full list of child healthcare centers is available at ClinicalTrials.govNCT04082689.

Swedish child healthcare is nurse-led, free of charge, available to almost everyone (99%) and focuses on preventive care [31]. The social insurance system in Sweden provides parental leave compensation for 480 days. Three months are exclusive to each parent respectively; the rest can be divided as the family decides. Day care services are available from 1 year of age and are available for all families as the cost is heavily subsidized. Of all children in Sweden, 80% attend day care by the age of 2 years [31]. At the time of the start of the study, the Swedish day care services do not have any general recommendations on toilet training or support in regular toilet habits.

### Internal pilot phase

The internal pilot phase of the full intervention was conducted over 12 months at one child healthcare center. The internal pilot phase started recruitment in April 2019. The aim was to evaluate study participant eligibility, recruitment rate, withdrawal rate and reason for withdrawal, completion of the web surveys and intervention adherence. Furthermore, it was important during this phase to gain knowledge of the child healthcare nurses’ acceptability of the intervention and recruitment, experienced work load and logistics of the study. After evaluation, only minor corrections were made in the study protocol to ensure data collection. The study participants in the pilot phase (*n* = 22) could therefore be included in the BABITT-study and will be retained in the full study analysis.

### Study population

The study enrolls full-term infants before they reach the age of 2 months and 2 weeks. Infants with malformations or disorders that may affect the gastrointestinal or urinary tract are excluded from participation as well as infants born small for gestational age (< 2 SD birth weight) or premature (< gestational week 37 + 0). Infants of parents with insufficient understanding of the Swedish language are not eligible; hence, the questionnaires are in Swedish.

### Description of intervention

The intervention consists of parents practicing assisted infant toilet training with their child.

Preceding the study start, the child healthcare nurses are given two 60-minute lectures on infant toilet training techniques and the BABITT-study structure and logistics. They receive a book, “Baby on the potty” [32], containing more elaborate descriptions of techniques and parental experiences of infant toilet training, as well as a brief summary brochure produced by the study researchers (Terese Nilsson (*TN*) or Anna Leijon (*AL)*.

At the regular meetings with the child healthcare nurses, parents are asked if they want to participate in the BABITT-study. If so, an introductory meeting is set up with one of the study researchers (*TN* or *AL)*. After receiving informed consent from both parents, group allocation is determined by randomization.

Parents of children being allocated to Group A are instructed to initiate toilet training as soon as possible. At the introductory meeting they receive a potty, oral instructions on how to conduct assisted infant toilet training, a book on the topic [32] as well as a brief summary brochure produced by the study researchers (*TN* and *AL*).

Parents of children allocated to Group B are instructed to start toilet training at the earliest at 9 months, but no later than at 11 months of age. Group B will receive the information and material as described above, at 9 months of age.

For further guidance, an educational parental forum was planned and held on a few occasions in 2019, but because of the corona pandemic, the educational parental forum had to be cancelled. Instead, if support or guidance is needed, a telephone contact with the study researchers is offered, in addition to the continuous guidance at the routine visits to the child healthcare centers.

In the event of reported infant colic, an infant behavioral diary is filled in by the parents (instructions given to both groups at inclusion). General recommendations are given by the child healthcare nurse, in accordance with The Swedish National Guide to Child Healthcare [33], to parents in both groups, in case of symptoms of infant colic. The general recommendations include routine examination by a family physician to rule out organic cause, a test period of milk-protein-free diet (in infant formulas or in mother’s diet if breastfeeding), testing probiotic containing *Lactobacillus Reuteri* and psychosocial support [33].

### Definition of assisted infant toilet training

The intervention, to be active in assisted infant toilet training, is defined by the making of at least one attempt a day (without the requirement of a successful outcome) on at least 5 out of 7 days per week.

### Intervention-adherence

In each web survey the parents of children allocated to Group A are asked to what extent they conduct infant toilet training. Parents of children allocated to Group B are asked in the web survey at 2, 3, 6 and 9 months of age, if they are following the group allocation, i.e. *not* undertaking assisted infant toilet training.

To ensure adherence to the intervention, the study researchers (*TN* or *AL*) contact parents of children allocated to Group A, if they state in the web survey at 2 months of age that they conduct infant toilet training *less* than 5 days per week. The parents are asked what obstacles they are facing and if they need support. If so, they receive extra information and individual support. The same procedure is made with children allocated to Group B, if stated at the 12 months web survey that the parents conduct assisted infant toilet training less than 5 days per week.

### Primary outcome

The primary outcome measure is the prevalence of functional gastrointestinal disorders (infant colic, infant dyschezia and/or functional constipation, defined according to ROME IV criteria) [17] up to the age of 9 months (Table 1), as parent-reported symptoms in the web surveys.

**Table 1:** The definitions of infant colic, infant dyschezia and functional constipation, according to the ROME IV criteria

**The ROME IV criteria:**	
**Infant colic**	
All of the following criteria are required:	
• Healthy infant ≤5 months of age at the onset and cessation of symptoms	
• Recurrent periods of prolonged crying, fussing, or irritability without obvious cause which cannot be prevented or resolved by parents	
• No evidence of infant failure to thrive, fever, or illness	
• Crying or fussing ≥3 hours during at least 3 days for a period of 7 days	
• 24-hour prospective behavioral diary documenting ≥3 hours crying/fussing^a^	
**Infant dyschezia**	
All of the following criteria are required:	
• Healthy infant ≤9 months of age at the onset and cessation of symptoms	
• At least 10 minutes straining and crying before successful or unsuccessful passage of soft stools	
**Functional constipation**	
At least 2 of the following criteria are required during a one-month period:	
• ≤2 defecations per week	
• History of excessive stool retention	
• History of painful or hard bowel movements	
• History of large-diameter stools	
• Presence of a large fecal mass in the rectum	
In toilet-trained children the following additional criteria may be used:	
• Stool incontinence at least once a week	
• History of large-diameter stools that may obstruct the toilet	

^a^Since there is no specific infant behavioral diary recommended by the ROME foundation, a modified infant behavioral diary is adapted from Landgren et al. [34] and is used in the BABITT-study

### Secondary outcomes

The secondary outcomes are the prevalences of functional gastrointestinal disorders (defined as constipation, gastrointestinal symptoms and/or stool toileting refusal) and urinary tract disorders (defined as bladder dysfunction and/or urinary tract infections) up to the age of 4 years, with the specific secondary outcome measures as described below:

1. **The prevalence of functional constipation** (defined according to ROME IV criteria) [17] will be evaluated up to 4 years of age (Table 1), as parent-reported symptoms in the web surveys.
2. **Gastrointestinal symptoms** will be measured with a Pediatric Quality of Life Inventory Gastrointestinal Symptoms Module (PedsQLGastro) [35] at 4 years of age. The Swedish validated version of PedsQLGastro is used [36]. PedsQLGastro contains 58 items. The items are reverse-scored and linearly transformed to a 0–100 scale (0 = 100, 1 = 75, 2 = 50, 3 = 25, 4 = 0), so that lower scores demonstrate more gastrointestinal symptoms, and hence, lower disease specific health-related quality of life. The scale scores are computed as the sum of the items divided by the number of items answered.
3. **The prevalence of stool toileting refusal** will be evaluated at the age of 4 years, as parent-reported symptoms in the web survey. Stool toileting refusal is defined as children who can urinate in the potty/toilet without difficulties, but refuse to have a bowel evacuation in the potty/toilet, during a period of more than 1 month [7].
4. **Rectal diameter**, as a complementary measure of functional constipation, will be measured by abdominal ultrasound at 9 months and 4 years of age [37]. A 60-day window is allowed (14 days before and 46 days after the due date) at 9 months, of age and a 120-day window is allowed (30 days before and 90 days after the due date) at 4 years of age. A rectal diameter > 30 mm is considered a sign of functional constipation at 4 years of age. The method is adapted from a manual by the University of Gothenburg (personal communication). To date, there is no knowledge of what is considered normal rectal diameter at the age of 9 months, but studies are ongoing (personal communication). An inter-intra-rater agreement analysis will be performed on the measurement results of the specialized staff of the BABITT-study.
5. **The prevalence of bladder dysfunction** will be evaluated at the age of 4 years, as parent-reported symptoms in the web surveys. For bladder dysfunction the ICCS terminology is used [19] and for diagnosis a bladder symptom questionnaire is used, validated in a Swedish population with a scoring system and cut-off for dysfunction [38].
6. **The prevalence of urinary tract infections** will be evaluated up to 4 years of age as parent-reported information in the web surveys. If stated, the medical record will be reviewed. Diagnosis of urinary tract infection is defined by the diagnostic numbers N.30.0 (acute cystitis), N30.9 (cystitis UNS) and N10.9 (acute pyelonephritis).
7. **Infant-to-mother attachment**, will be measured with the Maternal Postnatal Attachment Scale (MPAS) at 3 and 9 months [39]. The MPAS scale contains 19 items regarding mothers’ emotional response to their infants and their attachment. Each item is scored 1–5. Total score is calculated by adding scores on all items. Scale range for total score is 19–95. A higher score indicates higher infant-to-mother attachment.
8. **Parental stress** will be measured with the Swedish Parenthood Stress Questionnaire (SPSQ) at 3, 9 and 18 months and 2.5 years of age [40]. SPSQ is an adapted Swedish version of the Parental Stress Index [41]. It measures perceived stress in parenting in five dimensions (incompetence, role restriction, social isolation, spouse relationship and health problems) and contains 34 items, each scoring 1–5. Total score is calculated as an average of answers [1–5] on all items. Scale range for total score is 34–170. A higher score indicates higher parental stress.

### Additional data collection

1. **Data on intervention adherence and fidelity to allocation** will be collected until the toilet training process is completed, as parent-reported information in the web surveys.
2. **Socio-demographic data on parents** (age of the parents, country of birth, educational level, presence of siblings and family history of functional constipation) will be collected at baseline, as parent-reported information in the web survey.
3. **Data on participating children** (gender, weight at birth, gestational age at birth, breastfeeding and nutrition) will be collected up to 18 months of age, as parent reported information in the web surveys.
4. **Data on day care services** (the child’s attendance at day care service, and parental experience of the day care service’s provided support concerning potty training and toilet habits) will be collected up to 4 years of age, as parent-reported information in the web surveys.
5. **Parental experiences of the toilet training process** up to 2.5 years of age will be explored, as parent-reported experiences in the web surveys. The web surveys contain one item with a 6-point rating scale and a free text area.

### Participant timeline

**Fig. 1:**
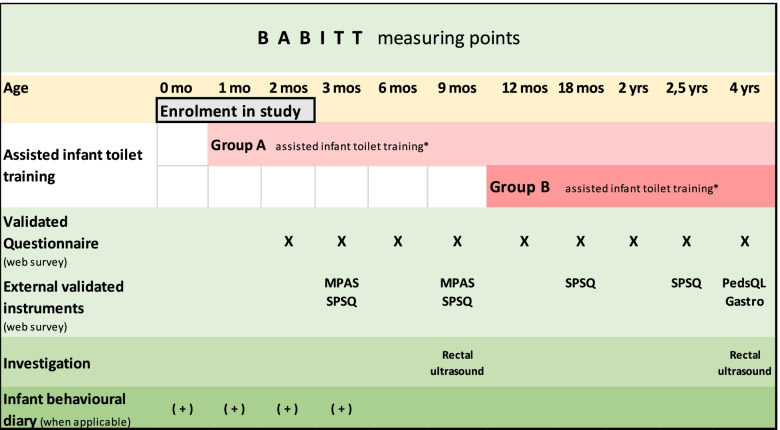
Time schedule over measuring points for the questionnaires (web surveys) (X) including the external validated instruments Maternal Postnatal Attachment Scale (MPAS), Swedish Parenthood Stress Questionnaire (SPSQ), Pediatric Quality of Life Inventory Gastrointestinal Symptoms Module (PedsQLGastro), and rectal ultrasound and infant behavioral diary (+) in the BABITT-study

Web surveys are conducted at 2, 3, 6, 9, 12, 18 months and at 2, 2.5 and 4 years (see Fig. 1). A 28-day window (defined as 14 days before and 14 days after the due date), is allowed for the 2, 3 and 6-month survey. At 9 months, a 44-day window is allowed (14 days before and 30 days after the due date) and at 12, 18 months and at 2 and 2.5 years a 60-day window is allowed (30 days before and 30 days after the due date). At 4 years of age a 120-day window will be allowed (30 days before and 90 days after the due date).

### Sample size estimation

The sample size was calculated on the estimated prevalence of functional gastrointestinal disorders (infant colic, infant dyschezia and/or functional constipation) up to 9 months of age. The prevalence of infant colic varies between 17 and 25% up to 6 weeks of age [15], and the prevalence of infant dyschezia varies between 0.9–3.9% [17]. However, at the time for sample size estimation, unpublished data from an ongoing Swedish cohort (personal communication) indicated a higher prevalence of infant dyschezia (18%) at 2 months of age and 4% at 6 months of age. Furthermore, the prevalence of functional constipation varies between 0.7–30% [23] and data from the same ongoing Swedish cohort indicated 7% prevalence of constipation up to 12 months of age (personal communication).

Thus, we have calculated that the total prevalence of functional gastrointestinal disorders in the general population (congruent with Group B in the BABITT-study) could be estimated to 15%, and that the prevalence in children who have been introduced to assisted infant toilet training (congruent with Group A in the BABITT-study) could be estimated to 4%.

In order to find a clinically relevant and statistically significant difference (*p* < 0.05) between groups with an 80% power, a number of 121 children are required in each study group. With an estimated 10% shortfall, 134 children will be included in each study group, which gives a total number of 268 children in the BABITT-study.

### Allocation/randomization

Participants are randomly assigned to either Group A or Group B with a 1:1 allocation after completion of baseline data in the electronic clinical research form (eCRF). A randomization program is provided and designed by MediCaseAB [42]. The randomization is stratified by the gender of the child using permuted blocks.

### Blinding

Due to the nature of the intervention, parents are informed to which group they are allocated. However, the group allocation (Group A or Group B) of the participating children will be blinded to the specialized staff performing the ultrasound measurement of rectal diameter at 9 months and 4 years of age.

### Data collection and management

The parent who is on parental leave at inclusion is asked to fill in all the web surveys up to 4 years of age. This will enable comparisons over time of each parent’s experience regarding the toilet training process, infant-to-mother attachment (MPAS) [39] and parental stress (SPSQ) [40].

The electronic web surveys are filled in directly by the parent on a smart phone, tabloid or computer, at the different time points during the study period. Thus ensuring data accuracy, user acceptability and timeliness of data receipt.

To enhance data collection, the study coordinator is alerted by the eCRF if the parent has not answered the web survey, and contacts the parent to encourage participation before the time window for the web survey is passed.

All items in the questionnaires (web surveys) were initially developed and validated according to Content Validity Index (CVI) [43], to ensure validity of data.

### Retention of participants

Regardless of adherence to the intervention, all participants will be included in an intention-to-treat analysis. Parents are encouraged to leave comments in a free text section in the web surveys, in case of non-adherence to the intervention.

Participants may withdraw from the study for any reason at any time. They are asked (but not obliged) to state the reason for withdrawing (according to GCP standards) on a form. It is clearly stated that withdrawal will not affect the future care given by the child healthcare centers. Participants may also be withdrawn if the study sponsor or government or regulatory authorities terminate the study prior to its planned end date.

All information collected in the BABITT-study will be electronically entered into the eCRF provided by MediCaseAB [42] and then securely stored in a database located in Sweden. At inclusion, study participants are given a specific study ID that is used for rectal ultrasound measurements and in the infant behavioral diaries.

Documentation of all changes in the eCRF is available via electronic logs and audit trails. Original data will be kept for 10 years after the last publication.

All data will be coded under data processing and publication of results. The code key will be destroyed when no longer needed, or 10 years after the last publication.

### Statistical methods

The results will be analyzed in accordance with intention-to-treat. Group A will be compared with Group B for all primary analyses up to the age of 9 months. For comparison of dichotomous variables between groups, the Chi-2 or Fisher’s extract test will be used, and for comparison of continuous variables, the Mann-Whitney U-test will be used. For correlation and agreement between scales, Pearson’s correlation test or Cohen’s kappa will be used when appropriate. Subsequently logistic regression analysis will be used to evaluate clinical outcome in relation to baseline data. For all tests, we will use 2-sided *p*-values with alpha ≤0.05 level of significance.

Quantitative longitudinal data will be analyzed statistically in conjunction with qualitative data from the questionnaires (mixed method).

## Discussion

The effect of assisted infant toilet training on functional gastrointestinal disorders (infant colic, infant dyschezia and/or constipation) and/or urinary tract disorders (bladder dysfunction and/or urinary tract infection) in young children has not previously been studied. If assisted infant toilet training can prevent functional gastrointestinal or urinary tract disorders, significant health benefits are to be expected due to reduced suffering for the children and their families, as well as a more effective use of healthcare resources. The conducting of infant toilet training has no known extra costs, and most likely reduces the use (and cost) of diapers.

The BABITT study also aims to explore parental experiences of assisted infant toilet training, and to evaluate if there is a difference between the intervention groups regarding mother-to-infant attachment and experienced parental stress.

The Swedish National Guide to Child Health Care stated in 2015 that the child healthcare nurses should encourage initiation of toilet training at the latest on the 10-months visit [14]. Therefore, it was unethical to recruit parents who would not receive any advice or intervention on toilet training in children older than 9 months. Consequently, in the BABITT-study, the control group consists of children who have *not* yet been introduced to toilet training during their first 9 months (Group B).

Furthermore, two reference materials, outside the BABITT-study, will be used as future comparators, comprising children who did *not* start toilet training during the first year of life.

One group comprises 110 healthy children in an ongoing longitudinal study in Gothenburg, regarding bowel and micturition habits in healthy children (personal communication). Infants were recruited from 2014 to 2019. These children have *not* been introduced to assisted infant toilet training during the first year of life and are thus suitable reference material for the BABITT-study.

Furthermore, a group of 4-year-old children in a cross-sectional study called BABIS-4 (Bowel and Bladder function In Swedish 4-year olds) will be useful for comparison. The BABIS-4 study is designed and planned by the research group (*TN, AL, US, ALH, BHS*) and will recruit 4-year old children during 2021–2022, at the same child healthcare centers as those participating in the present BABITT-study.

The questionnaires answered by parents in these two reference groups are congruent with the questionnaire (web survey) at 4 years of age in the BABITT-study.

There are practical issues pertinent in this study, particularly regarding the randomization of the study participants. Randomization enhances the scientific value of the results of the study and controls for unexpected confounders. The parents and the child healthcare nurses are informed that the study involves testing two different forms of assisted infant toilet training, and that both are considered equal options. Group A reflects how assisted infant toilet training is conducted in Vietnam [10] and Group B reflects how we introduced the potty in Sweden in the 60’s [2]. Group B is also in line with the current recommendations of starting toilet training not later than 10 months of age provided by The Swedish National Guide to Child Health Care [14].

It can be challenging to mobilize participants for intervention studies in lower socioeconomic groups. We have therefore chosen to recruit families from child healthcare centers in both rural and urban settings, with different distribution of socioeconomic standards. Data collection from several different child healthcare centers also reduces the risk of recruitment bias, facilitates participant recruitment and increases the generalizability of results.

As an effect of the corona pandemic, most introductory meetings with the parents and the study researchers (*TN* or *AL*) are held online as a video call. This facilitates parents being properly informed on the study and traveling to the child healthcare centers was avoided. To enhance participation, a low criterion for fidelity to the intervention is chosen (one attempt of assisted infant toilet training a day, 5 days per week). The dose-response rate (intensity) of assisted infant toilet training and outcome measures will be explored.

In summary, the BABITT study will evaluate whether assisted infant toilet training during the first year of life can prevent functional gastrointestinal and urinary tract disorders in children up to 4 years of age. If effective, new evidence-based guidelines could be implemented in child healthcare settings and at day care services.

## Trial status

Recruitment of study participants started in April 2019 and has continued throughout 2021. Data collection for the primary outcome will be completed when all children have turned 9 months (during 2022) and data collection for the secondary outcomes and follow-up for the whole study will be completed when all participants reach the age of 4 years  (2023–2025).

## Data Availability

To obtain the complete questionnaires of the BABITT-study, please contact study researchers (*TN*, *AL* or *BHS)*.

## Abbreviations

BABITT-study: Bowel And Bladder function in Infant Toilet Training study
BABIS-4: Bowel and Bladder function In Swedish 4-year olds
CRF: Clinical Research Form
eCRF: Electronic Clinical Research Form
MPAS: Maternal Postnatal Attachment Scale
PedsQLGastro: Pediatric Quality of Life Inventory Gastrointestinal Symptoms Module
SPSQ: Swedish Parenthood Stress Questionnaire
